# Associations between multimorbidity and unintentional falls among older adults with coronary heart disease

**DOI:** 10.3389/fpubh.2025.1698823

**Published:** 2025-11-27

**Authors:** Xuejie Qi, Xue Yao, Shanshan Liu, Li Fan, Xiaojuan Wu, Xiao Qi, Fuyan Shi, Suzhen Wang, Fuzhong Xue

**Affiliations:** 1 Key Laboratory of Medicine and Health of Shandong Province, School of Public Health, Department of Health Statistics, Shandong Second Medical University, Weifang, Shandong, China; 2 Department of Interventional Vascular Surgery, Zibo 148 Hospital, Zibo, Shandong, China; 3 Department of Cardiac Critical Care and Rehabilitation, Weifang People's Hospital, Weifang, China; 4 Department of Biostatistics, School of Public Health, Cheeloo College of Medicine, Shandong University, Jinan, Shandong, China

**Keywords:** coronary artery disease, multimorbidity, falls, older adults, fractures

## Abstract

**Objectives:**

Multimorbidity in individuals with coronary heart disease (CHD) is associated with an increased incidence of falls. We aimed to identify multimorbidity patterns and investigate the impact of different comorbidity patterns on fall and consequent fractures in older adults with CHD.

**Methods:**

We identified 189,558 older adults (aged ≥ 60 years) with CHD between January 2015 and December 2021 from the Shandong Multicenter Healthcare Big Data Platform. Multimorbidity patterns were identified through latent class analysis (LCA). Fine and Gray competing risk regression models were used to assess the associations between multimorbidity pattern and falls and fall-related fractures.

**Results:**

Four distinct multimorbidity clusters were observed: a gastrointestinal-dominant pattern (Class 1, 49.34%), a core cardiovascular disease pattern (Class 2, 15.50%), a metabolic-cardiovascular disease pattern (Class 3, 19.00%), and a cerebrovascular-osteoarticular complex pattern (Class 4, 13.60%). Older adults in Class 3 (HR = 1.19, 95% CI: 1.02–1.39), and Class 4 (HR = 1.68, 95% CI: 1.46–1.95) had an increased fall risk after confounder adjustment compared with those in Class 1, with mild comorbidities. The highest risk of falls with fractures (especially for sternum fractures) was found in older adults in Class 4. The risks of falls and falls with fracture were greater for females and patients aged ≥ 80 years.

**Conclusion:**

In this study, we analysis multimorbidity clusters among older patients with CHD and their association with fall and falls with fracture risk. We found that CHD patients with a cerebrovascular-osteoarticular complex pattern face the highest unintentional falls and fractures risk. Our findings can help stratify the risk of falls in older CHD patients and support precision public health initiatives.

## 1 Introduction

Coronary heart disease (CHD) is one of the most common chronic conditions and has been identified as a risk factor for both unexplained and recurrent falls (1). Falls, in turn, represent the leading cause of injury among older adults, often leading to accelerated functional decline, loss of independence, institutionalization, and premature mortality (2, 3). In CHD, impaired cardiac output and cerebral perfusion may contribute to postural instability, creating a vicious cycle of reduced physical activity, deconditioning, and increased fall risk. The consequences of falls, such as fractures, are more severe in individuals with CHD than in those without the disease (4).

Currently, over two-thirds of older patients with cardiovascular disease are reported to develop multimorbidity, which is defined as the coexistence of two or more chronic conditions within an individual (5, 6). This multimorbidity in CHD patients significantly impacts adverse outcomes such as falls, life expectancy and quality of life (7). Recent data have shown that older individuals with multimorbidity may face a substantially greater risk of falls (8, 9). Therefore, understanding multimorbidity patterns in specific populations is important for patient-oriented prevention, management, and prognosis.

Strength training emerges as a key strategy for healthy aging, as it not only prevents frailty and falls but also improves quality of life in older adults. Muscle strengthening, particularly in the lower limbs, reduces fall risk and supports balance and functional autonomy (10). Aging is a natural stage of life involving physical, psychological, and social changes. As people age, maintaining or improving quality of life is essential for active and healthy aging. Among non-pharmacological strategies, physical exercise is one of the most effective approaches to improving quality of life in old age (11).

Despite the high prevalence of multimorbidity among older adults with CHD, little is known about how specific comorbidity patterns influence the risk of falls and related fractures. Therefore, this study aimed to identify distinct multimorbidity patterns among patients with CHD and to examine their associations with unintentional falls and subsequent fractures.

## 2 Methods

### 2.1 Data source

The Shandong Multicenter Healthcare Big Data Platform (SMCHBDP) is a hybrid system developed by the Health Commission of Shandong Province in China developed in 2017. This big data platform employs a multistage sampling approach combining simple random, stratified, and cluster sampling methods, based on the population proportion and scale of different regions in Shandong Province. The platform includes approximately 5 million participants randomly selected from 42 representative counties across 16 cities in Shandong Province (12). After determining the research population, the ID numbers of the research population were encrypted and transformed into unique identifiers. Using this identity identifier as the sole index, the information from health-related databases such as individual electronic health records, electronic medical records, resident medical insurance payment systems, and death registration was merged. The platform followed up once a year since the enrolment of participants (finishing baseline filling date).

### 2.2 Case selection

In this study, the subjects were derived from the SMCHBDP. Inclusion criteria were: (1) registration in the SMCHBDP between January 1, 2015 and December 31, 2021; (2) age ≥ 60 years at enrolment; and (3) diagnosis of CHD (ICD-10 code I25.1). Exclusion criterion: history of falls prior to enrolment. During the study period, the research began with the patient’s first registration on the platform (i.e., no prior records exist for that individual within the study window), and ended when the patient experienced first occurrence of an unintentional fall, death, or study end (31 December 2021). Patient information, including demographic characteristics (sex, age, and residence location) and diagnostic information (diagnoses, symptoms, and past diseases) classified according to the International Classification of Diseases 10th revision (ICD-10) was extracted for analysis. CHD was identified via the ICD-10 codes I25.1. There were 189,558 patients with CHD in the final analysis.

The study was approved by the Ethics Committee of the School of Public Health, Shandong Second Medical University, China. All data were anonymized prior to analysis. Patients and the public were not involved in the design, conduct, reporting, or dissemination of this research. Researchers can only use the encrypted data on the SMCHBDP server after approval by the official review committee.

### 2.3 Exposures and outcomes

The following 31 chronic conditions were used as exposures in the present study: cardiovascular system diseases (including primary hypertension, angina pectoris, myocardial infarction, cardiac arrhythmias, heart failure, cerebral infarction, and cerebral atherosclerosis), respiratory system diseases (including chronic bronchitis, chronic obstructive pulmonary disease (COPD), asthma, and interstitial lung diseases), musculoskeletal system diseases (including arthropathies, spondylosis, cervical disk disorders, dorsalgia, soft tissue disorders, and osteoporosis), digestive system diseases (including peptic ulcers, chronic gastritis and duodenitis, non-infective enteritis and colitis, and chronic liver disease), blood diseases (including anemia), metabolic diseases (including hypothyroidism, goiter, type 2 diabetes, and dyslipidemia), mental system diseases (including depressive or anxiety and sleep disorders), eye diseases (including conjunctivitis and senile cataract), and genitourinary system diseases [including chronic kidney disease (CKD)]. Disease diagnoses and codes, was derived from the SMCHBDP according to the ICD-10. The corresponding ICD-10 codes for all chronic conditions are listed in Supplementary Table S1. The main study exposure was represented by comorbidity patterns derived as detailed in the statistical analysis section.

The primary outcome was unintentional falls, which were defined by ICD-10 codes: W00, W01, W05–W10, and W17–W19. The secondary outcome was fall-related, which were defined by the ICD-10 codes: S02, S12, S22, S32, S42, S52, S62, S72, S82, S92, T02, and T12.

### 2.4 Statistical analysis

Latent class analysis (LCA) was applied at baseline using 31 chronic conditions among CHD patients to identify homogeneous multimorbidity clusters. LCA was used to identify underlying multimorbidity patterns without prior assumptions about their structure (13). We started with a 2-class model and successively increased the number of latent models to 5. We summarized the model fitness indices, including the maximum log-likelihood, Akaike information criterion (AIC), Bayesian information criterion (BIC), sample size-adjusted Bayesian information criterion (SSABIC), and entropy (Supplementary Table S2). In principle, a smaller BIC and AIC and higher entropy indicate a better model, but clinical interpretability and the increasing tendency of the maximum log-likelihood simultaneously should be taken into consideration comprehensively. The improvement in model fit (log-likelihood) plateaued after the four-class solution, suggesting minimal gains beyond this point. The 4 latent class model was the optimum alternative.

Fine and Gray competing risk regression models were used to assess the associations between multimorbidity and unintentional falls, which accounted for the competing risks of non-fall cause death. Moreover, the associations of individual chronic conditions, multimorbidity pattern and the cumulative number of comorbidities with unintentional falls were evaluated via the same Fine and Gray competing risk regression models. The models subsequently included adjusting covariates including age, sex, residential location, medication count, use of drugs.

We used numbers and percentages to describe categorical variables, and mean and standard deviations (SDs) to describe normally distributed continuous variables. Categorical variables were compared via chi-square tests or Fisher’s exact tests to verify significant differences. Analyses were performed in R (version 4.2.2) using poLCA and cmprsk packages. All analyses and data visualizations were conducted via the R program. Statistical significance was defined as a two-sided *p*-value < 0.05.

## 3 Results

### 3.1 Characteristics of the study patients

For the period from January 1, 2015, to December 31, 2021, a total of 189,558 older patients with CHD were included in the study for analysis, of whom 103,459 (54.58%) were female, and 86,099 (45.42%) were male. The mean age was 72.12 years. Overall, approximately 2.56% of patients were without any comorbidities. The majority of patients (97.44%) had multimorbidity, with a maximum of 18 chronic conditions. Approximately 18.83% of these patients had 3 comorbidities in addition to CHD, with the highest proportion. Among the 189,558 patients, 1753 patients with were diagnosed for unintentional falls during follow-up. Compared with patients who were not diagnosed for fall during follow-up, those who falls were older (*p* < 0.0001), were more likely female (*p* < 0.0001), lived in urban areas (*p* < 0.0001), and had a greater proportion of five or more comorbidities (*p* = 0.0001) (Table 1).

**Table 1:** Descriptive characteristics of patients with CHD.

Characteristic	Total (*n* = 189,558)	Event during follow-up		
		Unintentional falls (*n* = 1753)	Not falls (*n* = 187,805)	*P*
Age (mean ± SD)	72.12 ± 8.36	74.89 ± 8.87	69.93 ± 8.14	<0.0001
Sex, *n* (%)				
Male	86,099 (45.42)	525 (29.95)	85,574 (45.57)	<0.0001
Female	103,459 (54.58)	1,228 (70.05)	102,231 (54.43)	
Age group, *n* (%)				
60 ~ 64	61,894 (32.65)	410 (23.39)	61,484 (32.74)	<0.0001
65 ~ 69	40,579 (21.41)	300 (17.11)	40,279 (21.45)	
70 ~ 74	32,358 (17.07)	298 (17.00)	32,060 (17.07)	
75 ~ 79	26,514 (13.99)	307 (17.51)	26,207 (13.95)	
80 ~ 84	17,385 (9.17)	259 (14.77)	17,126 (9.12)	
≥85	10,828 (5.71)	179 (10.21)	10,649 (5.67)	
Residential location, *n* (%)				
Urban	115,588 (60.98)	1,148 (65.49)	114,440 (60.94)	<0.0001
Rural	73,970 (39.02)	605 (34.51)	73,365 (39.06)	
Number of comorbidities, *n* (%)				
0	4,850 (2.56)	35 (2.00)	4,815 (2.56)	0.0001
1	15,417 (8.13)	149 (8.50)	15,268 (8.13)	
2	29,460 (15.54)	190 (10.84)	29,270 (15.59)	
3	35,691 (18.83)	215 (12.26)	35,476 (18.89)	
4	34,443 (18.17)	281 (16.03)	34,162 (18.19)	
5	26,412 (13.93)	247 (14.09)	26,165 (13.93)	
6	15,849 (8.36)	234 (13.35)	15,615 (8.31)	
7	9,149 (4.83)	191 (10.90)	8,958 (4.77)	
≥8	18,287 (9.65)	211 (12.04)	18,076 (9.62)	
Medication count				
≤5	63,728 (33.62)	410 (23.389)	63,318 (33.71)	<0.0001
≥6	125,830 (66.38)	1,343 (76.61)	124,487 (66.29)	
Use of antiplatelets	20,938 (11.05)	249 (14.20)	20,689 (11.02)	<0.0001
Use of anticoagulants	98,345 (51.88)	1,054 (60.13)	97,291 (51.80)	<0.0001
Use of nitrate drugs	136,283 (71.90)	1,309 (74.67)	134,974 (71.87)	0.014

**Figure 1:**
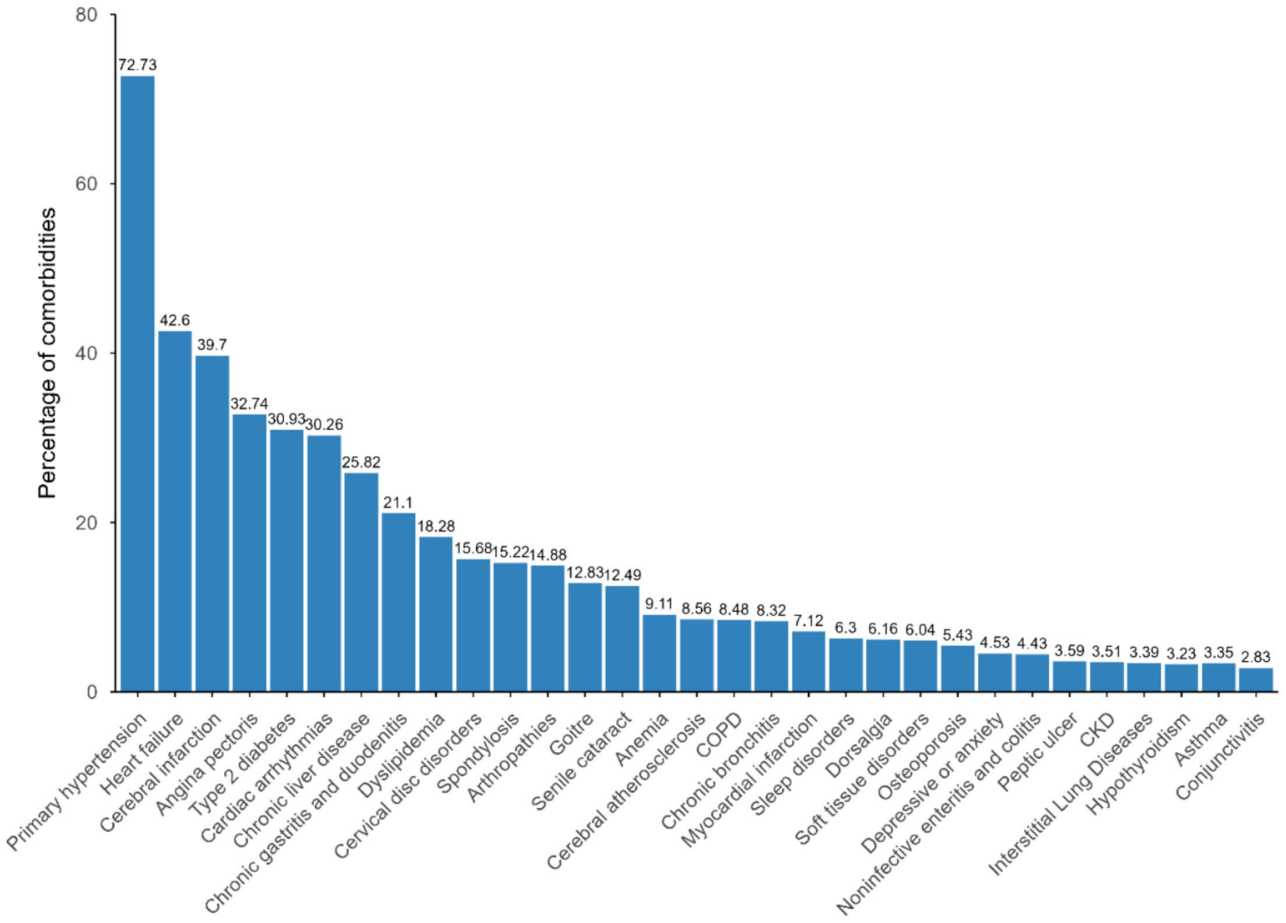
Distribution of choric conditions in patients with CHD.

The most prevalent multimorbidity was primary hypertension (*n* = 137,874, 72.73%), followed by heart failure (*n* = 80,751, 42.60%), and cerebral infarction (*n* = 75,262, 39.7%). In addition to cardiovascular diseases, a high percentage of patients were diagnosed with type 2 diabetes (*n* = 58,633, 30.93%), chronic liver disease (*n* = 48,948, 25.82%), and chronic gastritis and duodenitis (*n* = 40,006, 21.10%) (Figure 1).

### 3.2 Multimorbidity pattern in CHD patients identified by LCA

**Figure 2:**
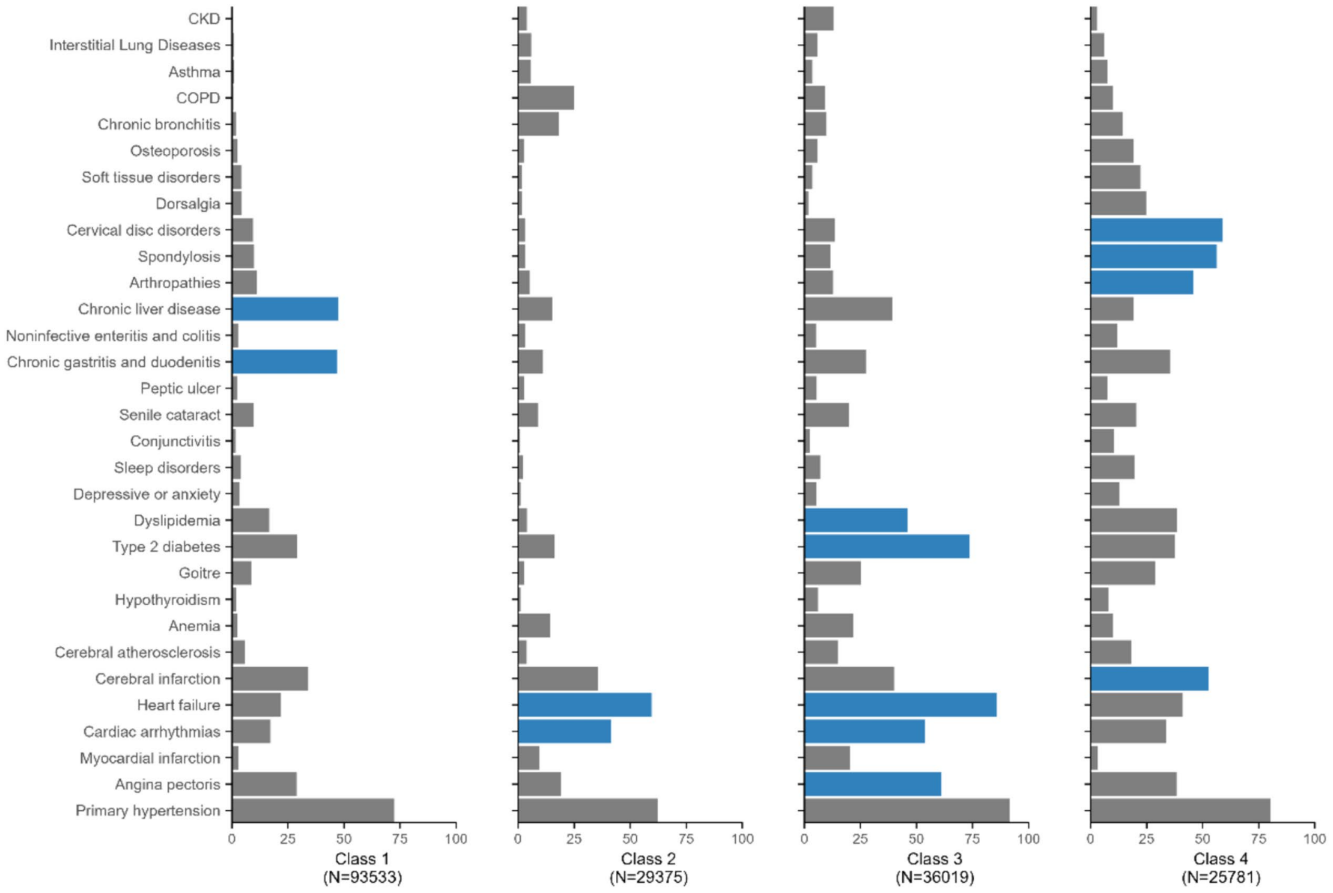
Prevalence (%) of each chronic condition within each latent class. The dark blue indicates the prevalence within class exceeds the prevalence among the all four classes combined.

Latent class analysis was applied to explore potential multimorbidity phenotype clusters in CHD patients. According to the model fitness indices, the four-class model with the optimal fit and the most reasonable clinical interpretability was ultimately selected (Supplementary Table S2 and Figure 2). Each class was labeled based on its chronic conditions whose prevalence within the class were higher than among the overall patients with multimorbidity. The four classes were the gastrointestinal-dominant pattern (*N* = 93,533, 49.34%), the core cardiovascular disease pattern (*N* = 29,375, 15.50%), the metabolic-cardiovascular disease pattern (*N* = 36,019, 19.00%) and the cerebrovascular-osteoarticular complex pattern (*N* = 25,781, 13.60%). The patients in class 1 had the highest prevalence of chronic liver disease (47.29%), followed by chronic gastritis and duodenitis (46.84). Class 2 was characterized by the highest prevalence of heart failure (59.58%), followed by cardiac arrhythmias (41.45%). Class 3 was characterized by the highest prevalence of heart failure (85.90%), followed by type 2 diabetes (73.63%), angina pectoris (61.01%), cardiac arrhythmias (53.83%), and dyslipidemia (46.09%). Class 4 was characterized by the highest prevalence of cervical disk disorders (58.75%), followed by spondylosis (56.23%), cerebral infarction (52.74%), and arthropathies (45.87%) (Supplementary Table S3). All conditions’ prevalences by class were showed in Supplementary Table S3.

The baseline characteristics by pattern are reported in Table 2. Patients in Class 3 were the oldest, with a mean age of 77.21 years and had the highest proportion of males (*N* = 22,222, 61.70%). The proportion of patients in Class 3 generally increased with age, but it generally decreased in Class 1, 2 and 4. In addition, the proportion of patients with ≥8 comorbidities was highest in Class 4 and the lowest in Class 1. There were more patients in Class 1 (*n* = 10,700, 11.4%) who lived with at most one chronic condition than those in Class 2 (*n* = 2,563, 8.73%), Class 3 (*n* = 2,154, 5.98%) and Class 4 (*n* = 0).

**Table 2:** Baseline characteristics stratified by multimorbidity cluster of patients with CHD.

Characteristic	Multimorbidity cluster			
	Class 1 (*N* = 93,533)	Class 2 (*N* = 29,375)	Class 3 (*N* = 36,019)	Class 4 (*N* = 25,781)
Age (mean ± SD)	67.12 ± 6.41	71.91 ± 7.93	77.21 ± 8.19	68.00 ± 7.13
Sex, *n* (%)				
Male	37,775 (40.39)	14,809 (50.41)	22,222 (61.70)	9,162 (35.54)
Female	55,758 (59.61)	14,566 (49.59)	13,797 (38.30)	16,619 (64.46)
Age group, *n* (%)				
60 ~ 64	40,452 (43.25)	6,482 (22.07)	2,844 (7.90)	10,375 (40.24)
65 ~ 69	23,722 (25.36)	5,719 (19.47)	4,156 (11.54)	5,804 (22.51)
70 ~ 74	15,318 (16.38)	5,889 (20.05)	5,947 (16.51)	4,453 (17.27)
75 ~ 79	9,209 (9.85)	5,740 (19.54)	7,918 (21.98)	3,132 (12.15)
80 ~ 84	3,805 (4.07)	3,694 (12.58)	8,091 (22.46)	1,434 (5.56)
≥85	1,027 (1.10)	1851 (6.30)	7,063 (19.61)	583 (2.26)
Region, *n* (%)				
Rural	53,651 (57.36)	22,537 (76.72)	18,529 (51.44)	18,899 (73.31)
Urban	39,882 (42.64)	6,838 (23.28)	17,490 (48.56)	6,882 (26.69)
Number of comorbidities, *n* (%)				
≤1	10,700 (11.44)	2,563 (8.73)	2,154 (5.98)	0 (0)
2–4	59,277 (63.38)	18,966 (64.56)	21,181 (58.80)	170 (0.66)
5–7	23,485 (25.11)	6,722 (22.88)	11,676 (32.42)	9,527 (36.95)
≥8	71 (0.07)	1,124 (3.83)	1,008 (2.80)	16,084 (62.39)

### 3.3 The risk of unintentional falls in CHD Patients with multimorbidity

**Figure 3:**
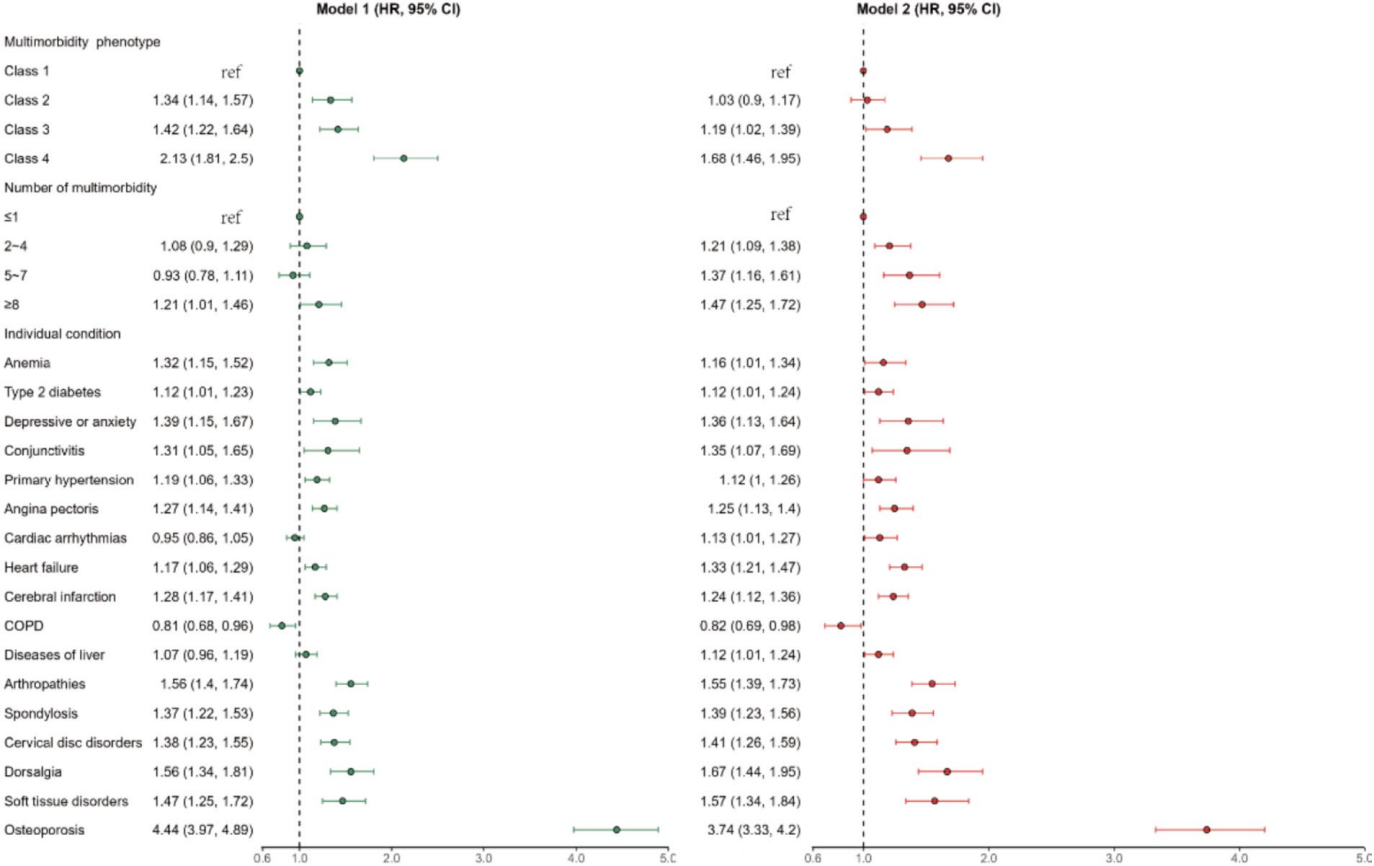
The HRs for falls associated with multimorbidity phenotype, number and individual conditions among older Chinese patients with CHD. Model 1 is adjusted for age, sex, residential location. Model 2 is adjusted for age, sex, residential location, medication count, use of antiplatelets, use of anticoagulants, use of nitrate drugs.

During follow-up, a total of 1753 patients experienced unintentional falls. The incidence rate of falls was 19.63 per 10,000 person/year with the highest rates observed among study patients in class 4 (28.16 per 10,000 person/year) (Supplementary Table S4). We further formulated a Fine and Gray competing risk regression model to evaluate the risks of falls in multimorbidity phenotype clusters, the number of comorbidities, and individual chronic conditions. Class 1, with the mild and fewest comorbidities, was the reference group. After adjustment for potential confounders, the patients in Class 2, Class 3, and Class 4 had a 1.03-fold [95% CI: (0.90, 1.17)], 1.19-fold [95% CI: (1.02, 1.39)] and 1.68-fold [95% CI:(1.46, 1.95)] increase in falls, respectively, compared with the patients in Class 1 (Figure 3).

An increased number of comorbidities was strongly associated with the risk of falls. Compared with those with ≤1 multimorbidity, those with 2–4 multimorbidity, 5–7 multimorbidity and ≥8 multimorbidity had adjusted HRs (95% CIs) of 1.21 (1.09, 1.38), 1.37 (1.16, 1.61) and 1.47 (1.25, 1.72), respectively (Figure 3). Patients with osteoporosis, dorsalis, and arthropathies had the highest unintentional fall risk, with adjusted HRs (95% CIs) of 3.74 (3.33, 4.20), 1.67 (1.44, 1.95) and 1.55 (1.39, 1.73), respectively (Figure 3).

Significant associations with an increased risk of falls and falls with fracture were stronger for women (Supplementary Table S5). With respect to age, we found that falls and falls with fracture risk were greater among individuals with the Class 2, 3 and 4 pattern who were ≥80 years (Supplementary Table S5).

### 3.4 Multimorbidity patterns and the risk of falls with fracture in CHD patients

In this study, we calculated the incidence rates of falls with fractures in different locations and analyzed the risk by multimorbidity patterns. The incidence rate of falls with femur fracture was 6.08 per 10,000 person/year, with the highest rates observed patients in class 3 (8.36 per 10,000 person/year). The incidence rates of falls with sternum fractures, and falls with lumbar spine and pelvis fractures were 3.90 and 3.57 per 10,000 person/year, respectively, with the highest rates observed patients in class 4 (8.24 and 7.81 per 10,000 person/year, respectively) (Supplementary Table S4).

After adjusting for potential confounders and compared with those of the class 1 pattern, the risk of falls with fractures increased by 21% (95% CI: 1.04–1.40) and 55% (95% CI: 1.36–1.77) in patients in Class 3 and Class 4, respectively. The patients in Class 2 (HR = 1.27, 95% CI: 1.03–1.61) and Class 3 (HR = 1.45, 95% CI: 1.17–1.89) had higher risks of falls with femur fractures. In addition, the patients in Class 4 had greater risks of falls with sternum fractures (HR = 2.49, 95% CI: 1.93–3.20), of falls with lumbar spine and pelvis fractures (HR = 2.74, 95% CI: 2.11–3.57), of falls with shoulder and upper arm fractures (HR = 1.43, 95% CI: 1.05–1.93), of falls with forearm and hand fractures (HR = 1.47, 95% CI: 1.03–2.11), of falls with forearm and hand fractures (HR = 1.39, 95% CI: 1.09–1.78), of falls with lower leg and foot fractures (HR = 1.56, 95% CI: 1.13–2.17) (Table 3).

**Table 3:** Association between multimorbidity patterns and falls-related injury.

Injury	Hazard ratio (95% confidence interval)		
	Class 2	Class 3	Class 4
Falls with fracture	1.11 (0.95, 1.29)	1.21 (1.04, 1.40)*	1.55 (1.36, 1.77)*
Falls with skull and facial bones fracture	0.68 (0.32, 1.43)	0.92 (0.49. 1.76)	1.24 (0.85, 1.79)
Falls with sternum fracture	0.81 (0.58. 1.15)	1.04 (0.75, 1.45)	2.49 (1.93, 3.20)*
Falls with lumbar spine and pelvis fracture	1.06 (0.78, 1.23)	1.17 (0.84, 1.64)	2.74 (2.11, 3.57)*
Falls with shoulder and upper arm fracture	0.72 (0.44, 1.17)	0.74 (0.45, 1.23)	1.43 (1.05, 1.93)*
Falls with forearm and hand fracture	1.12 (0.74, 1.69)	1.07 (0.72, 1.59)	1.47 (1.03, 2.11)*
Falls with femur fracture	1.27 (1.03, 1.61)*	1.45 (1.17, 1.89)*	1.39 (1.09, 1.78)*
Falls with lower leg and foot fracture	0.78 (0.49, 1.23)	1.08 (0.69, 1.71)	1.56 (1.13, 2.17)*

This model is adjusted for age, sex, residential location, revised as medication count, use of antiplatelets, use of anticoagulants, use of nitrate drugs. *p < 0.01.

## 4 Discussion

This study identified four distinct multimorbidity patterns among older adults with CHD and demonstrated that these patterns are differentially associated with unintentional falls and fractures. The findings highlight the complex interplay between cardiovascular, metabolic, musculoskeletal, and neurological systems in driving fall risk.

Our analysis of 189,558 CHD patients revealed that multimorbidity is common and strongly associated with fall risk. Hypertension, heart failure, diabetes, dyslipidemia, and cerebrovascular diseases were the most frequent comorbidities, consistent with prior studies (14, 15). Previous studies have demonstrated variable multimorbidity patterns among individuals with multiple cardiovascular diseases (16–18). Diverging from prior methodologies limited to 13–15conditions variables, our analysis systematically evaluated 31 chronic conditions (19). Beyond confirming known cardiovascular-metabolic clusters, our results uncovered novel patterns linking CHD with gastrointestinal and osteoarticular disorders. These multimorbidity configurations demonstrate differential fall risks, with the cerebrovascular-osteoarticular complex pattern conferring the highest risks of unintentional fall and fractures after fall, particularly among female.

While we have labeled the latent classes based on their most prevalent conditions, each cluster exhibits considerable internal heterogeneity, as shown in Supplementary Table S3. For example, within the “gastrointestinal-dominant” class, the presence of cerebral infarction introduces elements of possible motor impairment and cognitive deficit, compounding baseline fall risk (20, 21). Similarly, the co-occurrence of COPD in the “core cardiovascular disease” class may exacerbate functional decline through reduced exercise tolerance and muscle weakness (22). This multimorbidity complexity means that a uniform intervention strategy based solely on the dominant condition would be inadequate. Instead, a stratified approach is warranted: initial management should target the defining condition, but should be followed by systematic screening for high-risk comorbidities within that cluster. For example, patients in the “cerebrovascular-osteoarticular complex” class should be assessed not only for balance and gait deficits but also for dyslipidemia and diabetes, which can contribute to peripheral neuropathy and further impair stability (23, 24). Recognizing this intra-cluster heterogeneity is thus critical for designing precision interventions that address the full spectrum of an individual’s fall risk.

Physiological stresses resulting from the impairment of multiple organ systems in people with multimorbidity may synergistically increase vulnerability and risk for progressive morbidity and other adverse outcomes (25). Mechanistically, multimorbidity clusters drive pathogenesis through shared pathophysiological axes, notably via chronic low-grade inflammation (e.g., chronic inflammatory pathways in cardiovascular disease that potentiate type 2 diabetes progression and osteoarthritis pathogenesis) (26). Understanding the shared inflammatory and neurovascular pathways underlying multimorbidity may support the development of integrated interventions targeting both cardiovascular stability and musculoskeletal resilience. In addition, the research illuminates the crucial role of the bone-brain axis, where specific neuronal and molecular pathways contribute to postural instability (27). Furthermore, gait and balance deficits in older adults often overlap with chronic conditions that can cause transient cerebral hypoperfusion, a combination implicated in their increased risk of falls. This multimorbidity clustering not only amplifies fall susceptibility but also may precipitate significant mortality-morbidity burdens through fall-induced skeletal trauma (28). Hip fractures have emerged as the most consequential outcome, with excess lethality surpassing post-heart failure mortality (4, 29). In addition, biomechanically, thoracolumbar fractures of vertebral injuries, with burst fractures and compression fractures constituting the primary subtypes. This injury distribution reflects compounded osteoporosis and postural instability characteristic of advanced multimorbidity states (30, 31).

Our analysis revealed significant demographic disparities: fall patients presented an advanced age distribution and female predominance, which were correlated with elevated multimorbidity numbers. Age-related physiological decline and sex-specific factors, such as postmenopausal estrogen deficiency, likely contribute to the heightened fall risk observed among older female patients with multimorbidity (32–34). Compared with males, females are generally more prone to bone calcium loss because of their smaller and thinner bone structure, as well as the lack of protective effects of estrogen on the skeletal system (35). Geriatric CHD patients represent a clinically complex population that requires integrated management beyond cardiovascular care (36). Incorporating multimorbidity screening, particularly for musculoskeletal and cerebrovascular conditions, into routine cardiology practice could improve early fall’s risk identification. Targeted interventions focusing on balance, bone health, and medication optimization may meaningfully reduce fall incidence and improve overall prognosis.

Our study is subject to limitations inherent in its data sources, the lack of measurement for several established risk factors for falls, such as blood pressure variability, orthostatic hypotension, smoking and alcohol use history, and socioeconomic status (37, 38). Specifically, blood pressure variability and orthostatic hypotension are direct physiological triggers for dizziness and syncope, thereby increasing fall risk (39). Smoking and alcohol use serve as markers of overall health behavior and are associated with comorbidities such as COPD and peripheral neuropathy, which further elevate fall risk (40–42). If these factors are unevenly distributed between exposure groups and remain unadjusted for, the observed HR may overestimate the true effect, as some risk attributable to these confounders could be misattributed to the exposure. Similarly, lower socioeconomic status, encompassing economic disadvantage and limited social support, is a well-documented predictor of falls, partly due to constrained access to healthcare, poor nutrition, and hazardous living environments (43). If the exposed group had, on average, lower socioeconomic status than the unexposed group, this could also lead to overestimation of the HR. Therefore, the true association may be weaker than our point estimate suggests.

Although our study included key cardiovascular medications, it could not account for the pervasive effects of polypharmacy and other fall-risk-increasing drugs. For example, patients with cardiovascular and metabolic comorbidities are often prescribed a combination of antihypertensive and antidiabetic medications, which can cause orthostatic hypotension or hypoglycemia, thereby amplifying their inherent fall risk (24, 44). Similarly, those with cardiovascular and musculoskeletal conditions may use central nervous system-acting drugs like opioids or muscle relaxants, which directly impair balance and alertness (45, 46). Consequently, the hazard ratios for certain multimorbidity clusters may be confounded and potentially overestimated by this unmeasured medication burden. While our findings likely reflect this underlying synergy, future studies with comprehensive pharmacoepidemiologic data are needed to disentangle the specific contributions of drug exposures and disease clusters to fall risk.

The lack of data on several established risk factors for falls may have introduced residual confounding. Nevertheless, we analyzed adjusted HR using medication count as a proxy and discussed specific unmeasured confounders and theorizing the likely direction of the bias they introduce. In addition, the study’s large sample size and use of standardized electronic health records strengthen the robustness of our findings.

## 5 Conclusion

In summary, we identified four multimorbidity patterns among older adults with CHD, with the cerebrovascular-osteoarticular cluster conferring the highest risk of unintentional falls and fractures. These insights may inform precision prevention strategies and guide clinicians in implementing multidisciplinary interventions to mitigate fall-related morbidity and mortality. Future research should validate these patterns in diverse populations and evaluate targeted intervention models.

## Data Availability

The raw data supporting the conclusions of this article will be made available by the authors, without undue reservation.
