# Supplementary material for: Associations between multimorbidity and unintentional falls among older adults with coronary heart disease
Source: Front Public Health. 2025 Nov 27;13:1698823. doi: 10.3389/fpubh.2025.1698823 (PMC12695574; doi:10.3389/fpubh.2025.1698823)
Supplement: Supplementary file 1 [file Supplementary_file_1.docx]

**Supplementary materials**

Table S1 Chronic conditions with CHD patients and their ICD-10 codes

| ICD | disease | ICD | disease |
| --- | --- | --- | --- |
| D64 | Anemia | J41, J42 | Chronic bronchitis |
| E03 | Hypothyroidism | J44 | COPD |
| E04 | Goiter | J45-46 | Asthma |
| E11 | Type 2 diabetes | J80-84 | Interstitial Lung Diseases |
| E78 | Dyslipidemia | K27.4-27.7, K25.4-25.9, K26.4-26.9, K28.4-28.9 | Peptic ulcer |
| F32, 41 | Depressive or anxiety | K29.3, 29.4, 29.5 | Chronic gastritis and duodenitis |
| G47 | Sleep disorders | K50-52 | Noninfective enteritis and colitis |
| H10.4,10.5,10.8,10.9 | Conjunctivitis | K70-76 (except for K71) | Chronic liver disease |
| H25, H26 | Senile cataract | M00-25 | Arthropathies |
| I10 | Primary hypertension | M47-49 | Spondylosis |
| I20 | Angina pectoris | M50,51 | Cervical disc disorders |
| I22, 25.2 | Myocardial infarction | M54 | Dorsalgia |
| I44-49 | Cardiac arrhythmias | M60-79 | Soft tissue disorders |
| I50 | Heart failure | M80、81、82 | Osteoporosis |
| I63 | Cerebral infarction | N18 | CKD |
| I67.2 | Cerebral atherosclerosis |  |  |

Table S2 Fit Indices for Latent Class Models 1-4

| Number of classes | Log-likelihood | AIC | BIC | SSABIC | Entropy |
| --- | --- | --- | --- | --- | --- |
| 2 | -2003959 | 4008047 | 4008706 | 3980142 | 0.61 |
| 3 | -1981987 | 3964171 | 3965174 | 3831245 | 0.75 |
| 4 | -1971809 | 3943884 | 3945231 | 3792641 | 0.84 |
| 5 | -1964191 | 3928716 | 3930407 | 3779314 | 0.85 |

Table S3 Prevalences of chronic diseases among older CHD patients by class

| Diseases | Multimorbidity pattern | | | |
| --- | --- | --- | --- | --- |
|  | Class 1 | Class 2 | Class 3 | Class 4 |
| Anemia | 2.38 | 14.27 | 21.7 | 10.05 |
| Hypothyroidism | 1.95 | 1.01 | 6.05 | 7.82 |
| Goiter | 8.69 | 2.60 | 25.14 | 28.76 |
| Type 2 diabetes | 29.11 | 16.15 | 73.63 | 37.48 |
| Dyslipidemia | 16.67 | 3.88 | 46.09 | 38.41 |
| Depressive or anxiety | 3.46 | 1.02 | 5.25 | 12.95 |
| Sleep disorders | 4.01 | 2.27 | 7.06 | 19.54 |
| Conjunctivitis | 1.67 | 0.85 | 2.34 | 10.36 |
| Senile cataract | 9.68 | 8.81 | 19.9 | 20.34 |
| Primary hypertension | 72.39 | 62.39 | 91.52 | 80.1 |
| Angina pectoris | 28.82 | 18.96 | 61.01 | 38.35 |
| Myocardial infarction | 2.83 | 9.49 | 20.40 | 3.25 |
| Cardiac arrhythmias | 17.16 | 41.45 | 53.83 | 33.70 |
| Heart failure | 21.91 | 59.58 | 85.90 | 41.15 |
| Cerebral infarction | 34.07 | 35.65 | 40.01 | 52.74 |
| Cerebral atherosclerosis | 5.82 | 3.83 | 14.84 | 18.3 |
| Chronic bronchitis | 1.76 | 18.2 | 9.83 | 14.41 |
| COPD | 0.65 | 25.04 | 9.18 | 9.86 |
| Asthma | 0.99 | 5.73 | 3.57 | 7.65 |
| Interstitial Lung Diseases | 0.77 | 5.81 | 5.8 | 5.98 |
| Peptic ulcer | 2.24 | 2.71 | 5.46 | 7.58 |
| Chronic gastritis and duodenitis | 46.84 | 11.08 | 27.46 | 35.41 |
| Noninfective enteritis and colitis | 2.72 | 3.01 | 5.13 | 11.87 |
| Chronic liver disease | 47.29 | 15.26 | 39.4 | 19.25 |
| Arthropathies | 11.03 | 5.21 | 12.82 | 45.87 |
| Spondylosis | 9.89 | 3.13 | 11.57 | 56.23 |
| Cervical disc disorders | 9.4 | 2.97 | 13.71 | 58.75 |
| Dorsalgia | 4.21 | 1.76 | 1.75 | 24.77 |
| Soft tissue disorders | 4.15 | 1.57 | 3.57 | 22.26 |
| Osteoporosis | 2.5 | 2.52 | 5.91 | 19.26 |
| CKD | 0.33 | 3.86 | 12.89 | 2.90 |

Table S4 Incidence rate of injurious falls by multimorbidity pattern

| Injury | Incidence rate per 10 000 person/year | | | | |
| --- | --- | --- | --- | --- | --- |
|  | All | Class 1 | Class 2 | Class 3 | Class 4 |
| All falls | 19.63 | 17.20 | 20.53 | 18.00 | 28.16 |
| Falls with fracture | 16.09 | 14.18 | 14.12 | 16.93 | 23.50 |
| Falls with skull and facial bones fracture | 0.75 | 0.78 | 1.02 | 0.85 | 0.21 |
| Falls with sternum fracture | 3.90 | 2.94 | 4.01 | 2.61 | 8.24 |
| Falls with lumbar spine and pelvis fracture | 3.57 | 2.58 | 3.87 | 2.36 | 7.81 |
| Falls with shoulder and upper arm fracture | 1.67 | 1.63 | 1.29 | 1.09 | 2.58 |
| Falls with forearm and hand fracture | 2.03 | 2.08 | 2.24 | 0.67 | 3.30 |
| Falls with femur fracture | 6.08 | 4.76 | 7.14 | 8.36 | 6.52 |
| Falls with lower leg and foot fracture | 2.31 | 2.37 | 1.63 | 1.21 | 4.01 |

Table S5 The association between multimorbidity patterns and falls by sex and age

| Variable |  | Hazard ratio (95% Confidence Interval) | | |
| --- | --- | --- | --- | --- |
|  |  | Class 2 | Class 3 | Class 4 |
| **Sex** |  |  |  |  |
| Male | All falls | 0.98 (0.77, 1.31) | 1.00 (0.82, 1.32) | 1.44 (1.13, 1.85)^***^ |
|  | Falls with fracture | 1.00 (0.68, 1.49) | 1.03 (0.76, 1.55) | 1.48 (1.21, 1.97)^***^ |
| Female | All falls | 1.18 (0.91, 1.54) | 1.21 (1.05, 1.49)^**^ | 1.52 (1.23, 1.93)^***^ |
|  | Falls with fracture | 1.19 (0.87, 1.60) | 1.20 (1.01, 1.66)^*^ | 1.53 (1.22, 2.03)^***^ |
| **Age** |  |  |  |  |
| 60~69 | All falls | 0.86 (0.60, 1.24) | 1.00 (0.78, 1.27) | 1.55 (1.31, 1.85)^***^ |
|  | Falls with fracture | 0.92 (0.61, 1.37) | 1.05 (0.80, 1.36) | 1.53 (1.27, 1.85)^***^ |
| 70~79 | All falls | 0.75 (0.97, 1.59) | 0.93 (0.70, 1.25) | 1.32 (1.07, 1.63)^**^ |
|  | Falls with fracture | 0.74 (0.96, 1.66) | 0.93 (0.67, 1.28) | 1.32 (1.05, 1.66)^**^ |
| ≥80 | All falls | 1.23 (1.09, 1.68)^**^ | 1.31 (1.18, 1.68) ^***^ | 1.73 (1,26, 2.38)^***^ |
|  | Falls with fracture | 1.24 (1.06, 1.74)^**^ | 1.30 (1.15, 1.76) ^***^ | 1.88 (1.33, 2.66)^***^ |

Notes: The model is adjusted for age, sex, residential location, medication count, use of antiplatelets, use of anticoagulants, use of nitrate drugs. *P<0.05, **P<0.01, ***P<0.001.
